# The Nutritional Status of Chronic Obstructive Pulmonary Disease Exacerbators

**DOI:** 10.1155/2022/3101486

**Published:** 2022-10-13

**Authors:** Carina Rôlo Silvestre, Tiago Dias Domingues, Luís Mateus, Maria Cavaco, André Nunes, Ricardo Cordeiro, Teresa Silva Santos, Teresa Falcão, António Domingos

**Affiliations:** ^1^Serviço de Pneumologia, Centro Hospitalar do Oeste, Torres Vedras, Lisbon, Portugal; ^2^CEAUL, Faculdade de Ciências, Universidade de Lisboa, Lisbon, Portugal; ^3^Serviço de Nutrição, Centro Hospitalar do Oeste, Torres Vedras, Lisbon, Portugal

## Abstract

**Introduction:**

Malnutrition is underdiagnosed in chronic obstructive pulmonary disease.

**Objectives:**

This study aimed to evaluate the nutritional status of COPD patients and the link between dyspnea and nutritional status.

**Methods:**

This longitudinal observational study included patients hospitalized with exacerbated COPD. Nutritional status was assessed using Nutrition Risk Screening 2002, anthropometric, and biochemical assessments, in the first 48 hours of hospitalization.

**Results:**

Thirty patients were evaluated. According to the Nutrition Risk Screening 2002, half of the patients were at increased risk of malnutrition. 36.7% were classified as malnourished if we only considered the body mass index. From the evaluation of the tricipital skin fold, 69.0% were classified as malnourished, with 48.3% having severe malnutrition. According to the serum albumin level, 29.6% had malnutrition criteria. A significant association between dyspnea and increasing age (*p*=0.037) was found. There was a strong association between the fold classification and the degrees of severity of dyspnea (Fisher exact test: 13.60, *p*=0.001, *V* Cramer = 0.826). Most patients were malnourished and had higher grades of dyspnea. Tricipital skinfold reflects subcutaneous adipose tissue; this anthropometric measurement seems to be a good method to classify the nutritional status of COPD patients. It classified the biggest portion of patients as malnourished.

**Conclusion:**

The number of patients classified as malnourished changed with the method under analysis. The tricipital skin fold parameter was strongly associated with the dyspnea score. Most patients had adipose tissue and muscular mass depletion.

## 1. Introduction

Chronic obstructive pulmonary disease (COPD) is a prevalent and progressive disease with systemic involvement. Chronic inflammation is associated with lesions of the respiratory system. This correlates with multiple systemic comorbidities, such as high blood pressure, osteoporosis/osteopenia, gastroesophageal reflux, musculoskeletal dysfunction, ischemic heart disease, nutritional abnormalities, and others. Inflammatory mediators released in chronic respiratory diseases, such as COPD may induce anorexia [1–3].

In COPD, systemic inflammation is an important player in nutritional abnormalities and muscle dysfunction. Malnutrition is a serious problem in COPD patients. However, it is an underdiagnosed problem. Malnutrition impairs normal skeletal muscle functioning, with reduced muscle mass and decreased strength and endurance of respiratory muscles. Respiratory failure, then, becomes common as weight loss increases [4]. Malnutrition is associated with the severity of the disease, is related to more exacerbations, and increases hospital stays. Patients with advanced COPD are often seen with low body weight, and this has been recognized as a poor prognostic factor. The negative effect of malnutrition in COPD patients compromises health and quality of life. Thus, recognizing and treating malnutrition is crucial for overall patient management and optimizing outcomes [4–6]. There is no universal method for the diagnosis of malnutrition, but anthropometry measures and biochemical assessments may be helpful. Different studies use distinct methods, which makes it difficult to compare results.

## 2. Material and Methods

### 2.1. Study Design and Population

The authors conducted a longitudinal observational study with hospitalized exacerbated COPD patients from September 2019 to February 2020 in a Portuguese tertiary hospital.

All included patients had a previously established diagnosis of COPD according to postbronchodilator spirometry showing FEV1/FVC < 70%. The severity was classified according to GOLD criteria such as mild obstruction FEV1 ≥ 80%; moderate obstruction FEV1 ≥ 50%; severe obstruction FEV1 ≥ 30%; and very severe obstruction FEV1 < 30%.

Exacerbation was defined, according to GOLD, as an acute worsening of respiratory symptoms that needs additional therapy. All the included patients had severe exacerbations requiring hospitalization.

All the participants signed a written informed consent at the time of inclusion in the study.

### 2.2. Data Collection

Nutritional status was assessed using

(1) Nutrition Risk Screening 2002 (NRS-2002) (a patient with a score ≥3 is nutritionally at risk)
(2) Anthropometric measurements:
  - Body mass index (BMI) (BMI < 18.5 Kg/m^2^ was considered underweight, 18.5–25 kg/m^2^ normal weight, 25–30 kg/m^2^ overweight and > 30 kg/m^2^ obesity)
  - Midupper arm circumference (MUAC) and tricipital skinfold thickness (TST) were classified as malnutrition based on Frisancho reference-tables
(3) Biochemical: vitamin *D* (25-OH Vitamin *D* < 20 ng/mL was considered deficiency) and serum albumin (based on the criteria, mild malnutrition: 30 g/L ≤ albumin ≤34.9 g/L; moderate malnutrition: 25 g/L ≤ albumin ≤ 29.9 g/L; severe malnutrition: albumin < 25 g/L).

Assessments were made in the first 48 hours of hospitalization. All the anthropometric measurements were performed by the same trained dietitian.

### 2.3. Statistical Analysis

Statistical analyses were performed using SPPS software (version 23.0). Continuous variables are presented as mean ± standard deviation or median and respective interquartile range, depending on the normality underlying the data. For categorical variables, the respective absolute and relative frequencies are presented. The normality underlying the data was assessed using the Shapiro–Wilk test. The comparison of continuous variables that followed a normal distribution was performed using Student's *t*-test. For variables that have not followed a normal distribution the nonparametric Mann–Whitney test was used. Pearson's linear correlation coefficient was used as a measure of association between two continuous variables.

The significance value was taken as *p* < 0.05.

## 3. Results

A total of 30 patients were included in the study. Most of the patients were male 76.7% (*n* = 23), and 23.3% (*n* = 7) were female. The median age was 68 years, and the median BMI was 23.3 kg/m^2^.

Information on smoking habits was also collected, and it was found that 53.3% (*n* = 16) of the individuals were ex-smokers, and 43.3% (*n* = 13) were currently smoking. Only 3.3% (*n* = 1) were nonsmokers.

About 90% (*n* = 27) of our patients had three or more comorbidities. Older patients had more comorbidities (*r* = 0.424, *p*=0.020). According to GOLD criteria, about 43.3% (*n* = 13) of the patients had severe obstruction and 20% (*n* = 6) had a very severe obstruction. Most patients were medicated with triple bronchodilator therapy with a long-acting muscarinic antagonist, long-acting beta2-agonist, and inhaled corticosteroids. 66.7% (*n* = 20) had home oxygen therapy, and 40% (*n* = 12) patients had home noninvasive ventilation support. The demographic and clinical characteristics of COPD patients are presented in Table 1.

For these individuals, the number of exacerbations in the last year was recorded. 66.7% (*n* = 20) had two or more exacerbations without hospitalization, and 40% (*n* = 12) had at least one exacerbation with hospitalization. For 60% (*n* = 18) of the patients, this was their first severe exacerbation, with hospitalization.

At admission to the emergency department, half (*n* = 15) of the patients had global respiratory insufficiency, and 16.7% (*n* = 5) had respiratory acidemia. 40.0% (*n* = 12) were submitted to noninvasive ventilation support during hospitalization.

Regarding the application of the Modified Medical Research Council (mMRC) scale for dyspnea, the following results were observed: 6.7% (*n* = 2) classified as grade 1; 3.3% (*n* = 1) classified as grade 2; 43.3% (*n* = 13) as grade 3, and 30.0% (*n* = 9) as grade 4. No records were obtained for 16.7% (*n* = 5) of the individuals.

The authors found a significant association between dyspnea and increasing age (*p*=0.037).

Additionally, the authors proceeded to the evaluation of the nutrition status of individuals, taking into account different variables. The majority of patients (65%) had weight loss in the last 12 months. Half of COPD patients (*n* = 15) were at increased nutritional risk according to NSR-2002, and 36.7% (*n* = 11) were classified as malnourished according to BMI. From the evaluation of the TSF, 69.0% (*n* = 20) were classified as malnourished, with 48.3% (*n* = 14) having severe malnutrition. The midupper arm circumference (MUAC) classified 64% of the patients with malnutrition.

According to the serum albumin level, 29.6% (*n* = 8) had malnutrition criteria. There was no association between the severity degrees of dyspnea and malnutrition (*χ*^2^ = 1.264, *gl* = 2, *p*=0.796). It was found that almost all patients had vitamin D deficiency (*n* = 28, 93.3%).

In addition, there was no relationship between FEV_1_ (%) and the following variables: age (*r* = 0.143, *p*=0.583); BMI (*r* = 0.225, *p*=0.385); TST (*r* = 0.093, *p*=0.785); vitamin *D* (*r* = −0.534, *p*=0.074); albumin (*r* = −0.187, *p*=0.473); and MUAC (*r* = 0.409, *p*=0.211).

We also detected a strong association between skin fold and dyspnea (Fisher exact test 13.60; *p*=0.001, *V* Cramer 0.826).

Reclassifying patients into two categories mMRC ≤2 or ≥3, and comparing the different nutritional variables between groups, showed that patients with more dyspnea (mMRC ≥ 3 have a lower MUAC value ($\bar{\chi }$ = 23.2, SD = 5.5) than patients with less dyspnea complaints (mMRC ≤2 ($\bar{\chi }$ = 34.5, SD = 3.5) (*p*=0.001).

None of the patients needed to be transferred to the intensive care unit, and none died during hospitalization. The median hospital stay was 12 days (IQR 9.75). We observed that the time of hospitalization increases in patients with lower serum albumin levels (*r* = −0.491, *p*=0.015) and with lower BMI (*r* = −0.421, *p*=0.045).

## 4. Discussion

Assessing malnutrition and acting to promote an adequate nutritional status is crucial to the management of patients with COPD [5]. There is no gold standard method to assess the nutritional status of patients with COPD. BMI is a simple method for assessing nutritional status, and a low BMI is associated with a worse prognosis [7]. However, BMI may not accurately reflect the nutritional status of COPD patients [8]. Our results show that only a part of the patients was classified as malnourished if we used this measure.

The NSR-2002 was developed by the European Society for Clinical Nutrition and Metabolism and is widely used to assess nutritional status [7, 9].

In addition to BMI and NRS-2002, serum albumin is considered a good biomarker to evaluate nutritional status. In our study, this biomarker classified as malnourished only a small portion of our patients. Nutritional evaluation of COPD patients should not be limited to BMI or albumin level.

The body mass is divided into two parts: fat-free mass (FFM) and fat mass (FM). Fat mass is an energy store. FM and FFM decreases are important prognostic factors in COPD [4, 8, 10, 11].

The TST reflects subcutaneous adipose tissue as MUAC reflects muscle mass. In our study, FM and FFM given, respectively, by TST and MUAC classified the biggest portion of patients as malnourished (69% vs. 64%). FM and FFM depletion was present in the biggest portion of our patients. These two parameters seem to be useful to classify COPD exacerbators on their nutritional status. TST and MUAC were associated with the dyspnea score, reflecting the advanced stage of the disease. We also found that older patients had more comorbidities and more dyspnea.

Exacerbations are associated with an increased inflammation status, anorexia, and increased work of breathing. This has a direct impact on muscle dysfunction, causing an increased risk of hospitalization due to exacerbations. The loss of muscle mass compromises the work of the respiratory muscles, leading to a negative functional impact in these patients [12]. Showing that acute exacerbations have a negative impact on nutritional status and muscle function. Our patients were frequent exacerbators, and they had a significant loss of adipose tissue as well.

Cachexia is a complex metabolic syndrome characterized by involuntary and progressive loss of skeletal muscle mass with variable loss of fat mass. Adipose tissue in cachexia plays an important role in disease progression [13, 14]. The advanced disease has a more gradual loss of muscle and fat mass, as seen in our patients. The occurrence of multiple exacerbations leads to disease progression and depletion of adipose reserves. According to previous studies, the loss of muscle mass as well as of fat mass might be related to specific effects of hypoxia on energy balance [13].

We identified that COPD patients with lower levels of albumin, or lower BMI had prolonged hospitalization.

Anthropometric measures seem to be a poorly sensitive marker of mild-to-moderate nutritional abnormalities [12, 13]. This shows that our patients are at high nutritional risk, with severe nutritional abnormalities.

Several studies report that vitamin D deficiency contributes to severe COPD by magnifying inflammation. Vitamin D supplementation may have an effect in reducing exacerbations and is also important to comorbidities associated with COPD as osteoporosis [15–17]. Our results reflect that almost all of the COPD exacerbators had a Vitamin D deficiency.

More nutritional studies should be done assessing the different stages of COPD disease. May adipose tissue depletion be a therapeutic target?

## 5. Conclusion

Most of the patients had significant complaints of dyspnea in a stable phase of the disease. Dyspnea was higher in older patients. The number of patients classified as malnourished changed with the method under analysis. Nutrition screening in COPD exacerbators, only with BMI, leads to under diagnosis of malnutrition. The TST and MUAC assessment tools encompassed more patients in the undernutrition category and were strongly associated with the dyspnea score. These anthropometric measurement methods seem to be the best to stratify the nutritional status of COPD patients compared with the other methods under analysis, they allowed classifying a higher number of patients as malnourished. Vitamin D deficiency was prevalent in COPD exacerbators' patients. The majority of COPD exacerbators had adipose tissue and muscular mass depletion.

Nutritional status evaluation is an important parameter in COPD exacerbators. Once, by identifying the existence of malnutrition, we can treat it.

## Figures and Tables

**Table 1:** General characteristics of COPD patients.

All patients, *n* = 30	
Gender, *n* (%)	
Female	7 (23.3)
Male	23 (76.7)
Age, year, median (IQR)	68 (11)
Tobaco (pack-year)	50.8 ± 22.8
Smoking status, *n* (%)	
Current smokers	13 (43.3)
Ex-smokers	16 (53.3)
Never smokers	1 (3.3)
GOLD, *n* (%)	
A	1 (3.3)
B	7 (23.3)
C	0
D	22 (73.3)
Obstruction, *n* (%)	
FEV_1_ ≥ 80%	0
FEV_1_ ≥ 50%	11 (36.7)
FEV_1_ ≥ 30%	13 (43.3)
FEV_1_ < 30%.	6 (20)
Bronchodilator therapy, *n* (%)	
LAMA + LABA	6 (20)
LAMA + LABA + ICS	24 (60)
Home non-invasive ventilation, *n* (%)	12 (40)
Home oxygen therapy, *n* (%)	20 (66.7)
Comorbidity, *n* (%)	
Hypertension	24 (80.0)
Diabetes	9 (30.0)
Dyslipidemia	18 (60.0)
Auricular fibrillation	7 (23.3)
Ischemic heart disease	9 (30.0)
Tuberculosis sequelae	5 (16.7)
Gastroesophageal reflux disease	5 (16.7)
Anxiety	5 (16.7)
Depression	4 (13.3)
Enphysema	19 (63.3)
Height, cm, median (IQR)	163 (6.50)
Weight, kg, mean ± SD	61.6 ± 19.6
BMI, Kg/m^2^, mean ± SD	22.7 ± 7.9
BMI, *n* (%)	
<18.5 Kg/m^2^	11 (36.7)
18.5–25 kg/m^2^	5 (16.7)
25–30 kg/m^2^	7 (23.3)
>30 kg/m^2^	7 (23.3)
NRS-2002, *n* (%)	
1	6 (20.0)
2	9 (30.0)
3	9 (30.0)
4	3 (10.0)
5	3 (10.0)
Albumin, g/L, median (IQR)	3.8 (0.8)
Total proteins, mean ± SD	6.0 ± 0.6
Vitamin D, ng/mL, mean ± SD	8.3 ± 2.6
MUAC, cm, mean ± SD	23.5 ± 7.2
TST, mm, mean ± SD	7.6 ± 4.2

LAMA: long-acting muscarinic antagonist; LABA: long-acting beta2-agonist; ICS: inhaled corticosteroids; BMI: body mass index; MUAC: midupper arm circumference; TST: tricipital skinfold thickness.

## Data Availability

The data used to support the findings of this study are available from the corresponding author upon request.

## References

[1] Global Strategy for Prevention (2022). Diagnosis and Management of COPD 2022 Report. https://goldcopd.org/wp-content/uploads/2021/12/GOLD-REPORT-2022-v1.1-22Nov2021_WMV.pdf.

[2] Gea J., Sancho-Muñoz A., Chalela R. (2018). Nutritional status and muscle dysfunction in chronic respiratory diseases: stable phase versus acute exacerbations. *Journal of Thoracic Disease*.

[3] Dubé B. P., Laveneziana P. (2018). Effects of aging and comorbidities on nutritional status and muscle dysfunction in patients with COPD. *Journal of Thoracic Disease*.

[4] Mete B., Pehlivan E., Gülbaş G., Günen H. (2018). Prevalence of malnutrition in COPD and its relationship with the parameters related to disease severity. *International Journal of Chronic Obstructive Pulmonary Disease*.

[5] Rawal G., Yadav S. (2015). Nutrition in chronic obstructive pulmonary disease: a review. *Journal of Translational Internal Medicine*.

[6] Keogh E., Mark Williams E. (2021). Managing malnutrition in COPD: a review. *Respiratory Medicine*.

[7] Ingadottir A. R., Beck A. M., Baldwin C. (2018). Two components of the new ESPEN diagnostic criteria for malnutrition are independent predictors of lung function in hospitalized patients with chronic obstructive pulmonary disease (COPD). *Clinical Nutrition*.

[8] Raad S., Smith C., Allen K. (2019). Nutrition status and chronic obstructive pulmonary disease: can we move beyond the body mass index?. *Nutrition in Clinical Practice*.

[9] Chen R., Xing L., You C. (2016). Nutritional risk screening 2002 should be used in hospitalized patients with chronic obstructive pulmonary disease with respiratory failure to determine prognosis: a validation on a large Chinese cohort. *European Journal of Internal Medicine*.

[10] Rutten E. P., Calverley P. M. A., Casaburi R. (2013). Changes in body composition in patients with chronic obstructive pulmonary disease: do they influence patient-related outcomes?. *Annals of Nutrition and Metabolism*.

[11] Luo Y. W., Zhou L. Q., Li Y. (2016). Fat-free mass index for evaluating the nutritional status and disease severity in COPD. *Respiratory Care*.

[12] Gea J., Barreiro E. (2019). Nutritional abnormalities and chronic obstructive pulmonary disease. *International Journal of Tuberculosis & Lung Disease*.

[13] Schols A. M. W. J., Broekhuizen R., Weling-Scheepers C. A., Wouters E. F. (2005). Body composition and mortality in chronic obstructive pulmonary disease. *The American Journal of Clinical Nutrition*.

[14] Vegiopoulos A., Rohm M., Herzig S. (2017). Adipose tissue: between the extremes. *The EMBO Journal*.

[15] Jolliffe D. A., Greenberg L., Hooper R. L. (2019). Vitamin *D* to prevent exacerbations of COPD: systematic review and meta-analysis of individual participant data from randomised controlled trials. *Thorax*.

[16] Cook R., Thomas V., Martin R., NIHR Dissemination Centre (2019). Can treating Vitamin D deficiency reduce exacerbations of chronic obstructive pulmonary disease?. *British medical journal*.

[17] Kokturk N., Baha A., Oh Y. M., Young Ju J., Jones P. W. (2018). Vitamin D deficiency: what does it mean for chronic obstructive pulmonary disease (COPD)? a compherensive review for pulmonologists. *The Clinical Respiratory Journal*.

